# Supplementary material for: Effects of Catalyst Pretreatment on Carbon Nanotube Synthesis from Methane Using Thin Stainless-Steel Foil as Catalyst by Chemical Vapor Deposition Method
Source: Nanomaterials (Basel). 2020 Dec 28;11(1):50. doi: 10.3390/nano11010050 (PMC7824306; doi:10.3390/nano11010050)
Supplement: Supplementary file 1 [file nanomaterials-11-00050-s001.pdf]

Supplementary Material

Effects of catalyst pre-treatment on carbon nanotube synthesis from methane using thin stainless-steel foil as catalyst by chemical vapor deposition method

Thuan Minh Huynh [1,*], Sura Nguyen [1], Ngan Thi Kim Nguyen [1], Huan Manh Nguyen [1], Noa Uy Pham Do [1], Danh Cong Nguyen [2], Luong Huu Nguyen [1], Cattien V. Nguyen [3]

(a)

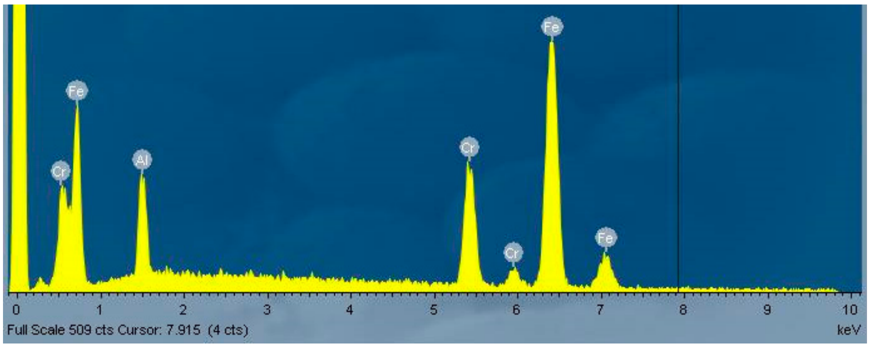

(b)

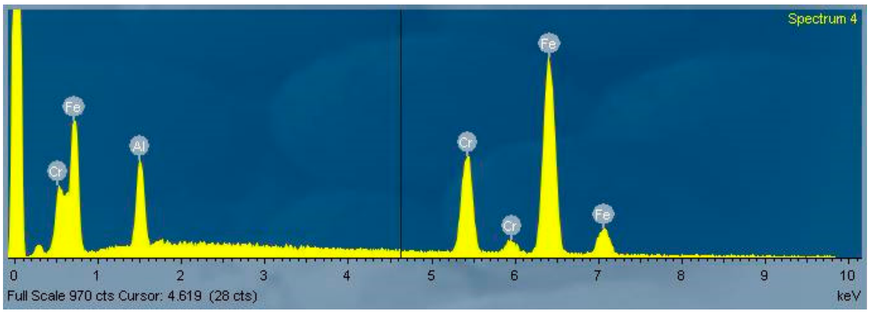

(c)

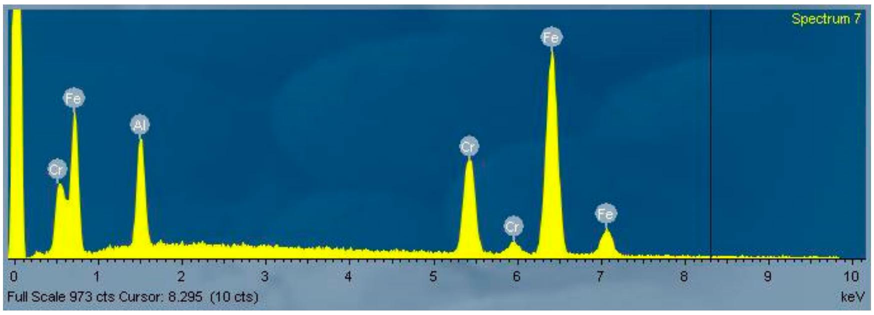

(d)

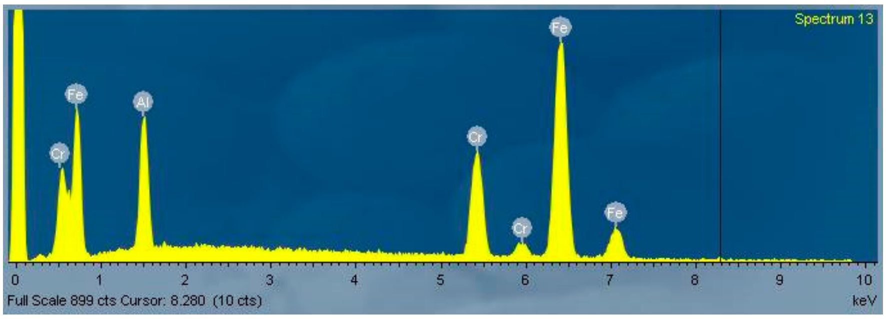

Figure S1. EDX spectrum of the catalyst precursor (a) and after pretreatment in 10 min at different temperatures: 750 ^{o}C (b); 850 ^{o}C (c) and 950 ^{o}C (d).
